# Effect of the COVID‐19 pandemic on surgical oncology practice—Results of an SSO survey

**DOI:** 10.1002/jso.26839

**Published:** 2022-03-06

**Authors:** Yelizaveta Gribkova, Catherine H. Davis, Alissa A. Greenbaum, Shou‐en Lu, Adam C. Berger

**Affiliations:** ^1^ Rutgers Robert Wood Johnson University Medical School New Brunswick New Jersey USA; ^2^ Division of Surgical Oncology Rutgers Cancer Institute of New Jersey New Brunswick New Jersey USA; ^3^ Department of Biostatistics Rutgers University School of Public Health New Brunswick New Jersey USA

**Keywords:** COVID‐19, multidisciplinary care, surgical oncology, telemedicine

## Abstract

**Background and Objectives:**

The COVID‐19 pandemic significantly affected healthcare delivery, shifting focus away from nonurgent care. The aim of this study was to examine the impact of the pandemic on the practice of surgical oncology.

**Methods:**

A web‐based survey of questions about changes in practice during the COVID‐19 pandemic was approved by the Society of Surgical Oncology (SSO) Research and Executive Committees and sent by SSO to its members.

**Results:**

A total of 121 SSO members completed the survey, 77.7% (94/121) of whom were based in the United States. Breast surgeons were more likely than their peers to refer patients to neoadjuvant therapy (*p* = 0.000171). Head and neck surgeons were more likely to refer patients to definitive nonoperative treatment (*p* = 0.044), while melanoma surgeons were less likely to do so (*p* = 0.029). In all, 79.2% (95/120) of respondents are currently using telemedicine. US surgeons were more likely to use telemedicine (*p* = 0.004). Surgeons believed telemedicine is useful for long‐term/surveillance visits (70.2%, 80/114) but inappropriate (50.4%, 57/113) for new patient visits.

**Conclusion:**

COVID‐19 pandemic resulted in increased use of neoadjuvant therapy, delays in operative procedures, and increased use of telemedicine. Telemedicine is perceived to be most efficacious for long‐term/surveillance visits or postoperative visits.

## 1 INTRODUCTION

Over the last 2 years, the COVID‐19 pandemic dramatically changed the focus and delivery of health care. In March of 2020, when coronavirus infections began increasing exponentially in the United States, nonurgent and elective procedures were suspended.^1^, ^2^ Only emergent and urgent surgical operations were allowed to proceed as to minimize the burden on surgical staff and the use of personal protective equipment and hospital beds. As the pandemic persisted, diagnosis and treatment of chronic conditions yielded to prevention of virus transmission and management of COVID‐19 patients.^3^ Unfortunately, cancer screening and select surgical management of cancer patients were also categorized as elective and subjected to delays and cancellations. Many surgical oncologists were forced to alter their practices to comply with the restrictions.^2^, ^4^, ^5^ The demotion of cancer management to “nonurgent,” combined with global stay‐at‐home recommendations, resulted in lower screening rates of melanoma, breast, colorectal, and lung cancers, fewer cancer diagnoses of all types, and a decrease in all cancer‐related patient encounters.^6^, ^7^, ^8^, ^9^ Additionally, while institution‐specific increased use of neoadjuvant therapy has been documented in breast, melanoma, and gastrointestinal (GI) cancers, the prevalence of increased neoadjuvant use across the field of surgical oncology has not yet been quantified.^4^, ^5^, ^10^

As the pandemic continued to burden the healthcare system, many needs in surgical oncologic care were met with the increased use of telemedicine. Telemedicine, which improves access to care and decreases healthcare costs for patients, has been shown effective and safe in a substantial portion of visits for patients with cancer specifically.^11^ However, the management of surgical oncologic patients differs from medical oncology in its surgical component, and from other surgical specialties in that cancer patients are more likely to require longitudinal, collaborative care. While many surgical specialties, such as dermatologic and endocrine surgery, found that telemedicine is appropriate in outpatient and postoperative patient management, the use of telemedicine in surgical oncology has not been analyzed.^12^, ^13^ As the first systemic survey of SSO members, this study aims to examine the effects of the COVID‐19 pandemic on the practice of surgical oncology, including current telemedicine practices for surgical oncology patients.

## 2 MATERIALS AND METHODS

Following approval by the Rutgers Cancer Institute of New Jersey Institutional Review Board, a voluntary web‐based survey was administered by the Society of Surgical Oncology to its active members via email yielding 121 responses. The survey collected demographic data from respondents, such as age, gender, country and state of practice, academic practice environment, current role, primary area of surgical oncologic practice, and years in practice. Members were also surveyed on the influence of COVID‐19 on their outpatient clinical and surgical practices, specifically clinic hours, operative schedules, office support staff, and use of telemedicine. Case volumes, effect of telemedicine on compensation, opinions on the appropriate use for and barriers to the use of telemedicine were queried. Information on changes in clinical practices, such as increased use of neoadjuvant therapy or use of nonoperative therapy was also gathered. Finally, respondents were asked if their institution had a virtual telemedicine platform before the pandemic and asked about pre‐pandemic or current telemedicine use. Estimates of patient volumes seen virtually were gathered during three distinct time periods—March to July 2020, August to December 2020, and January to May 2021. The participants were asked to rate the effectiveness of telemedicine using a 3‐point scale (more effective, less effective, about the same) and identify which type of visits they found to be appropriate for telemedicine (initial or new patient visit, postoperative visit, long‐term follow‐up surveillance visit).

Study data were collected and managed using REDCap electronic data tool provided by the Rutgers Cancer Institute of New Jersey. Participants were surveyed via email between May 6, 2021, and September 8, 2021, with an initial email followed by two reminder emails. Data collection occurred over 6 months with the final download in November 2021.

The data were analyzed using descriptive statistics. Two‐tailed Pearson's *χ* ^2^ tests and odds ratio (OR) were used to assess the relationship between surgical oncologic specialty or practice type and the use of neoadjuvant therapy, definitive nonoperative treatment, and deferment of operative procedures, as well as the relationship between practice type and decreased clinic hours, decreased operative procedures, covering nonspecialty shifts and COVID‐19 units, and changes in schedules. Two‐tailed Pearson's *χ* ^2^ tests were also used to compare the use of telemedicine and perceived effectiveness based on physician's age, stage of career, and use of telemedicine before the pandemic. Data were analyzed using Microsoft Excel version 16.55 (2021). Statistical significance was defined by a *p* value < 0.05.

## 3 RESULTS

### 3.1 Demographics, clinical operations, and clinical practices

Of the 121 respondents, the majority were practicing surgical oncologists (87.6%, 106/121) while the remainder of respondents were fellows, residents, and basic science researchers. Most respondents were between the ages of 35 and 64 (90.9%, 109/121) in various stages of their career. Comprehensive demographics for participants are listed in Table 1. Most respondents practice in an NCI‐designated Comprehensive Cancer Center with Academic Affiliation (44.9%, 53/118) or an Academic Institution without NCI‐designated Cancer Center (23.7%, 28/118). Most practices were located in the United States (77.7%, 94/121) though 14 other countries were also represented in the survey. Practices in urban areas were predominant in this survey (87.6%, 106/121), with only 0.8% (1/121) of respondents representing a rural practice. Of US respondents, 29 states were represented; most respondents were from the Northeast (40.9%, 38/93), followed by the Midwest, the Southeast, the West, and the Southwest (Table 1).

**Table 1:** Demographics of survey participants

Age	
<35	3 (2.5%)
35–44	42 (34.7%)
45–54	35 (28.9%)
55–64	32 (26.4%)
65 or older	9 (7.4%)
Gender	
Male	75 (62.0%)
Female	43 (35.5%)
Other	3 (2.5%)
Current role	
Surgical oncologist	106 (87.6%)
Fellow	6 (5.0%)
Resident	2 (1.7%)
Basic science researcher	3 (2.5%)
Other	4 (3.3%)
Years in practice	
Still in training	6 (5.0%)
Early career (<10 years)	37 (31.1%)
Mid‐career (10–25 years)	41 (35.3%)
Late career (>25 years)	34 (28.6%)
Practice setting	
NCI‐designated Comprehensive Cancer Center with Academic Affiliation	53 (44.9%)
NCI‐designated Cancer Center with Academic Affiliation	12 (10.2%)
Academic Institution without NCI‐designated Cancer Center	28 (23.7%)
Medical System/Nonacademic Hospital	17 (14.4%)
Multispecialty group practice	5 (4.2%)
Solo/independent practice	1 (0.8%)
Other	2 (1.7%)
Primary area of practice	
Breast	59 (48.8%)
Colorectal	22 (18.2%)
Endocrine	11 (9.1%)
GI	38 (31.4%)
GU	4 (3.3%)
Head and neck	11 (9.1%)
Hepatobiliary	29 (24.0%)
Melanoma	45 (37.2%)
Sarcoma/Musculoskeletal	40 (33.1%)
Thoracic	6 (5.0%)
Location of practice	
United States	94 (77.7%)
Outside of the United States	27 (22.3%)
Location of Practice in the US	
Midwest	19 (20.4%)
Northeast	38 (40.9%)
Southeast	15 (16.1%)
Southwest	8 (8.6%)
West	13 (14.0%)
Practice environment	
Rural (population < 5000)	1 (0.8%)
Suburban (population 5000–99,999)	14 (11.6%)
Urban (100,000+)	106 (87.6%)
COVID vaccinated	
Yes	115 (95.8%)
No	2 (1.7%)
Decline to answer	3 (2.5%)

Abbreviation: GI, gastrointestinal; GU, genitourinary

Most participants reported several changes to their practice and compensation during the COVID‐19 pandemic (Table 2). One‐half (49.6%, 60/121) of respondents reported more use of telehealth during the pandemic. Many saw substantial changes to their clinical (67.8%, 82/121) and operative (61.2%, 74/121) schedules, decreased operative procedures (45.5%, 55/121), and decreased clinical hours (27.3%, 33/121). Additionally, providers noted that there was a significant reduction in support staff (43.8%, 53/121) and clinic volume. Half of respondents (50.8%, 61/120) reported an 11%–50% decrease in clinical patients and a quarter (23.3%, 38/120) reported a 11%–25% decrease in operative patients. Many practices (22.3%, 27/121) instituted pay cuts; however, only 10.7% (13/121) of respondents were compensated lower than normally and only 2.5% (3/121) were not compensated at all. These changes were not dependent on the practice type or practice setting. These rapid and unpredictable changes correlated with perceived stress of the physicians. Three‐quarters (77.5%, 95/121) of surgical oncologists reported that their stress levels were either somewhat or very much increased due to the COVID‐19 pandemic (Table 2),

**Table 2:** Impact of COVID‐19 on clinical operations

Decreased clinic hours		
Yes		33 (27.3%)
No		88 (72.7%)
Decreased operative procedures in specialty		
Yes		55 (45.5%)
No		66 (54.5%)
Worked non‐specialty call shifts or on COVID units		
Yes		21 (17.4%)
No		100 (82.6%)
Increased number of telemedicine visits		
Yes		60 (49.6%)
No		61 (50.4%)
Changes in operative schedule		
Yes		74 (61.2%)
No		47 (38.8%)
Perceived reduction in volume of clinical patients		
0%		21 (17.5%)
1%–10%		33 (27.5%)
11%–25%		42 (35.0%)
26%–50%		19 (15.8%)
51%–75%		2 (1.7%)
>75%		3 (2.5%)
Perceived reduction in volume of operative patients		
0%		37 (30.8%)
1%–10%		38 (31.7%)
11%–25%		28 (23.3%)
26%–50%		10 (8.3%)
51%–75%		5 (4.2%)
>75%		2 (1.7%)
Clinic schedule substantially affected		
Yes		82 (67.8%)
No		39 (32.2%)
Reduced support staff in office		
Yes		53 (43.8%)
No		68 (56.2%)
Reduced physician staff in office		
Yes		14 (11.6%)
No		107 (88.4%)
Instituted pay‐cuts		
Yes		27 (22.3%)
No		94 (77.7%)
Currently being compensated at a lower rate		
Yes		13 (10.7%)
No		108 (89.3%)
Currently not being compensated		
Yes		3 (2.5%)
No		118 (97.5%)
Plan to retire due to the pandemic		
Yes		2 (1.7%)
No		119 (98.3%)
Stress levels		
Very much decreased		1 (0.08%)
Somewhat decreased		3 (2.5%)
Unchanged		22 (18.2%)
Somewhat increased		67 (55.4%)
Very much increased		28 (23.1%)

The type of care surgical oncologists decided to offer to cancer patients was also transformed by the pandemic (Table 3). Most surgical oncologists (53.7%, 65/121) reported that they were more likely to refer patients for neoadjuvant therapy than before the pandemic. Notably, 75% (3/4) of genitourinary surgeons, 71.2% (42/59) of breast surgeons, and 63.6% (7/11) of endocrine surgeons reported increased use of neoadjuvant therapy, with breast surgeons being more likely than other specialists to implement the practice (OR: 4.2, *p* < 0.000171). Most respondents deferred nonurgent cancer operations (71.1%, 86/121). Genitourinary surgeons (66.7%, 4/6), head and neck surgeons (62.5%, 5/8), melanoma surgeons (57.7%, 15/26), and sarcoma surgeons (52.4%, 11/21) had the highest rates of deferring nonurgent procedures until operating room restrictions were eased. Few surgeons performed a procedure in the office in lieu of the operating room (17.4%, 21/121). However, melanoma (OR: 5.8, 95% confidence interval [CI]: 2.06–16.48, *p* < 0.001), sarcoma (OR: 2.7, 95% CI: 1.03–7.03, *p* = 0.039), and head and neck (OR: 4.9, 95% CI: 1.33–17.96, *p* = 0.007) surgeons were significantly more likely to do so. Head and neck surgeons tended to be more likely than others to refer patients to definitive nonoperative therapy (OR: 4.2, 95% CI: 0.95–18.71, *p* = 0.059), while melanoma surgeons tended to be less likely to opt for nonoperative therapy (OR: 0.13, 95% CI: 0.02–1.08, *p* = 0.059). However, only 9.9% (12/121) of respondents referred patients to definitive nonoperative therapy (Table 3). There was no significant difference between practice types and implementation of neoadjuvant therapy, nonoperative treatment, performing procedures in the office, or deferring a nonurgent cancer operation.

**Table 3:** Changes in practice

Primary area of practice			Increased use of neoadjuvant therapy (%)	Increased referral to definitive nonoperative treatment (%)	Increased deferral of operative treatment (%)	Performing a procedure in the office (%)
Breast			71.2***	10.2	33.3	16.9
Colorectal			54.5	13.6	25.0	13.6
Endocrine			63.6		40.0	18.2
GI			47.4	7.9	33.3	15.8
GU			75.0		66.7	100
Head and Neck			54.5	27.3	62.5	45.5**
Hepatobiliary			51.7	13.8	27.3	10.3
Melanoma			51.1	2.2	57.7	33.3***
Sarcoma/Musculoskeletal			45.0	2.5	52.4	27.5*
Thoracic			33.3			

Abbreviations: GI, gastrointestinal; GU, genitourinary.

*p < 0.05; **p < 0.01; ***p < 0.001

Additionally, the survey found that multidisciplinary tumor boards largely moved to a completely virtual platform (91.7%, 111/121). As of now, 44.4% (52/117) of surgical oncologists prefer virtual tumor board to in person, while 32.5% (38/117) do not notice major differences between a virtual versus in‐person meeting platform.

### 3.2 Telemedicine

One of the significant impacts of the COVID‐19 pandemic on the delivery of health care has been a dramatic shift to telemedicine. While 45.8% (55/120) of the respondents had an institutional telemedicine platform before the pandemic, only 14.5% (17/117) were performing telemedicine personally. As of May 2021, 79.2% (95/120) of surgical oncologists were performing telemedicine; this trend was highest in academic institutions without NCI‐designated cancer centers (85.7%, 24/28) and at NCI‐designated comprehensive cancer centers with academic affiliation (84.9%, 45/53) followed by NCI‐designated cancer centers with academic affiliation (75%, 9/12) and nonacademic hospitals (64.7%, 11/17). US surgeons were more likely to use telemedicine than non‐US surgeons (*p* = 0.004). Hepatobiliary surgeons (89.7%, 26/29) and gastrointestinal surgeons (89.8%, 33/38) were most likely to use telemedicine, while only 40% (2/6) of thoracic surgeons reported the use of telemedicine. The increased use of telemedicine was not significantly influenced by physician's age, stage in career, or use of telemedicine before the pandemic (Table 4).

**Table 4:** Efficacy of telemedicine in surgical oncology

		Perception of efficacy of telemedicine independent of current usage		
	Currently using telemedicine (%)	Less effective (%)	Unchanged (%)	More effective (%)
Primary area of practice				
Breast	79.7	64.4	20.3	11.9
Colorectal	72.7	68.2	22.7	4.5
Endocrine	63.6	45.5	45.5	
GI	86.8	57.9	15.8	21.1
GU	75.0	75.0		
Head and Neck	72.7	90.9		
Hepatobiliary	89.7	58.6	17.2	20.7
Melanoma	71.1	53.3	33.3	11.1
Sarcoma/Musculoskeletal	82.5	50.0	32.5	12.5
Thoracic	40.0	83.3	16.7	
Use of telemedicine prior to before pandemic				
Yes	94.1	70.6	17.6	11.8
No	76.0	57.0	24.0	16.0
Practice setting				
NCI‐designated Comprehensive Cancer Center with Academic Affiliation	84.9	52.8	28.3	17.0
NCI‐designated Cancer Center with Academic Affiliation	75.0	41.7	25.0	25.0
Academic Institution without NCI‐designated Cancer Center	85.7	60.7	25.0	14.3
Medical System/Nonacademic Hospital	64.7	58.8	17.6	17.6
Multi‐specialty group practice	20.0	100		
Solo/independent practice	100	100		
Other	50.0	50.0	50.0	
Years in Practice				
Still in training	83.3	16.7	50.0	33.3
Early career (<10 Years)	86.5	54.1	32.4	13.5
Mid‐career (10‐25 years)	71.4	57.1	21.4	14.3
Late career (>25 years)	76.5	70.6	14.7	14.7
Location				
United States	84.0**	54.3	26.6	18.1
Outside of the United States	59.3	76.0	16.0	8.0

Abbreviation: GU, genitourinary.

**p < 0.01

One‐half of surgical oncologists (45%, 54/120) report that their patient volume for telemedicine was 11%–50% in March to July of 2020 and in 2021. However, almost two‐thirds (58.8%, 70/119) report the same volume for telemedicine patients in August to December of 2020.

The majority of surgical oncologists believe telemedicine is less effective than traditional patient care (59.3%, 70/118). However, those who are currently using telemedicine were more likely to consider it to be efficacious (*p* = 0.027). Billing was an issue cited by 42.1% (51/121) of respondents. Additionally, many (54.5%, 66/121) thought that access to technology for the patient was a significant barrier to telehealth. However, most physicians did not cite physician willingness to participate (78.5%, 95/121), patient willingness (71.1%, 86/121), or physician access to technology (86.8%, 105/121) as a reason they view telemedicine as less effective. Cited reasons for why telemedicine was less efficacious than traditional medicine was the inability to perform a physical exam, more insurance obstacles when seeing patients from a different state, and more administrative complications in insurance coverage and reimbursement. Some physicians felt that telemedicine impairs the physician's rapport with the patient and decreases the involvement of trainees, thereby detracting from their education. The perceived efficacy of telemedicine was not significantly influenced by the physicians' age or stage of career.

Importantly, one‐half of surgical oncologists (50.4%, 57/113) believe that telemedicine is an inappropriate tool for establishment of a new patient in their practice. However, 66.3% (63/95) of surgeons currently using telemedicine believe that it is an effective tool in management of established patients.

## 4 DISCUSSION

The COVID‐19 pandemic dramatically affected the practice of surgical oncology, altering the established patterns of treatment. As many cancer operations were deemed nonurgent and postponed, surgical oncologists were faced with difficult decisions on how to mitigate the potential harm caused by these delays. At the first systemic survey of SSO members, this study aimed to assess how SSO members adjusted their practice to manage the ramifications of the pandemic. This study found increased use of neoadjuvant therapy, increased deferral of nonurgent cancer operations, and increased use of telemedicine.

Most of the respondents (53.7%, 65/121) reported increased use of neoadjuvant therapy during this time. Most breast surgeons surveyed (71.2%, 42/59, *p* < 0.0001) increased their clinical use of neoadjuvant therapy, while this was only true for 33.3% (2/6) of thoracic surgeons. This may be due to the fact that breast cancer has the ability to achieve pathological complete response on systemic therapy and often has a measurably better prognosis with the use of neoadjuvant therapy.^10^, ^14^ However, lung and esophageal cancers, although also good responders to neoadjuvant therapy, have a lower survival rate with surgical delay.^15^ The increased use of neoadjuvant therapy was not dependent on the type of practice.

An overwhelming majority of respondents (71.1%, 86/121) in this study deferred nonurgent cancer operation during the pandemic; however, only 9.9% (12/121) of respondents referred patients for definitive nonoperative therapy. Melanoma surgeons were statistically less likely than others (*p* = 0.029) to refer their patients to nonoperative therapy not only because the definitive therapy for localized melanoma is surgery but also because melanoma is an aggressive cancer that, if not treated operatively in early stages, metastasizes to many organs.^16^ Melanoma (*p* = 0.001), sarcoma (*p* = 0.039), and head and neck (*p* = 0.01) surgeons were significantly more likely to perform a procedure in the office than other specialties. This is likely due to the existence of less invasive procedures for some of those cancers than for others, such as hepatobiliary or thoracic. The increase in the number of delayed procedures is not dependent on whether the practice was an NCI‐designated Cancer Center or not, nor whether the setting of the practice was urban, suburban, or rural. A large study recently published by the COVIDSurg collaborative which pooled data from 466 centers in 61 countries showed that delays in cancer surgery were prevalent worldwide at the beginning of the pandemic, with length of delay positively correlating with severity of lockdown measures by country.^17^

Timing surgery and neoadjuvant therapy for surgical oncology patients during the time of COVID‐19 is a balancing act. Patients undergoing neoadjuvant chemotherapy, which can cause immunosuppression, as well as surgery, when infected with SARS CoV‐2 have worse outcomes, higher infection rates, and increased intensive care unit (ICU) admissions.^18^, ^19^ In fact, cancer patients, in general, have increased postoperative admissions to the ICU, implying that when ICUs are full nationwide due to COVID‐19, scheduling a major cancer surgery could be unwise.^20^ On the other hand, delaying surgery can cause tumor progression or create emergent situations, such as bowel obstruction or spinal cord impingement, resulting in a more complex procedure and a higher risk of complications and death.^21^ A model created by the Canadian Partnership Against Cancer suggests that delaying cancer surgery by more than 6 weeks is likely to be detrimental to the survival of the patient.^21^ As the pandemic continues to threaten the scheduling of operations, surgical oncologists should continue to assess their patients individually, while aiming to not delay surgery by longer than absolutely necessary.

Rampant SARS CoV‐2 infections among patients and physicians alike, reduced support staff, and stay‐at‐home orders precipitated an unprecedented increase in the use of telemedicine.^22^ However, the efficacy of telemedicine as a comprehensive tool in surgical oncology is yet to be examined. One of the aims of this study was to assess current telemedicine practices for surgical oncology patients and their efficacy as perceived by physicians. While only 14.5% (17/117) of surgical oncologists surveyed in this study were performing telemedicine before the pandemic, almost 80% (95/120) of these surgeons are now using telemedicine in their practice. This study found that most surgical oncologists find telemedicine to be less effective than traditional office visits and inappropriate as a ubiquitous form of management for all surgical oncologic patients. While established or surveillance patients can be appropriately managed virtually, new patient visits and most postoperative patients require in‐person consultations. Respondents noted that telemedicine provides no opportunity for a thorough physical examination, such as a comprehensive skin, breast, and lymph node examination, which is of paramount importance in cancer screening and diagnosis. Similarly, postoperative patients, who require examination to ensure they are meeting their postoperative milestones, cannot be given adequate care virtually. Necessary patient workup, such as imaging and biopsy, cannot be done via a virtual appointment and can therefore delay cancer diagnosis and subsequent treatment. Respondents noted a perceived increase in the incidence of advanced stage breast cancer at the time of the first appointment which they attributed to lack of adequate physical exam. Supporting evidence for this observation can be found in the simultaneous decrease in diagnoses of cancers, such as breast, colorectal, and endometrial, during the Spring 2020 lockdown stage of the pandemic and increase in incidence of cancers found at a later stage post lockdown.^6^, ^7^, ^9^ As many cancer diagnoses are aided by physical exam findings, the practice of telemedicine in new patient visits in surgical oncology can pose a serious threat to the timeliness and effectiveness of treatment.

However, as telemedicine is likely a permanent fixture in all of medicine, surgical oncology included, there must be a method for standardization of the virtual physical examination to increase effectiveness. Orthopedic surgeons created a photo‐based standardized physical examination protocol that has similar accuracy to in‐person physical examinations.^23^ If a collaborative effort was made by SSO members to create a similar protocol for different cancer subtypes, perhaps the same parity between virtual and in‐person examination can be achieved.

Cancer can be a longer and a more emotionally arduous diagnosis than many other surgical conditions, therefore patients can benefit from a dependable relationship with their physician. This study found that many surgical oncologists feel that telemedicine does not allow them to build an adequate rapport with their patients. Congruently, a study in medical oncology found that some cancer patients felt reluctant to communicate with their doctors via a video platform due to feelings of self‐consciousness, and the absence of a physical examination.^24^, ^25^ As the perceived empathy of the provider and the patient's anxiety about their cancer can significantly impact the quality of life and oncologic outcome, the lack of rapport could prove to be a great barrier for telemedicine in surgical oncology.^26^ Conversely, the burden of expensive oncologic treatments can be mitigated by telemedicine which saves patients on travel expenses, time lost from work, and other related costs.^27^, ^28^

The members of SSO noted that there are technological and regulatory obstacles to telemedicine. Members believe that many of their patients do not have access to technology necessary to conduct a telehealth visit, and that there are insurance coverage and reimbursement barriers to seeing a patient from a different state. This was further exacerbated by strict Health Insurance Portability and Accountability Act of 1996 (HIPAA) guidelines outlining the necessary parameters for medical video communication.^29^ However, many of the regulatory and reimbursement guidelines have been curtailed due to the COVID‐19 pandemic. The Centers for Medicare and Medicaid Services (CMS) issued a waiver in March of 2020, which allowed telemedicine visits to have the same reimbursement rates as in‐person visits and extended the telemedicine benefits of original Medicaid enrollees.^30^ Additionally, HIPAA regulations surrounding telehealth were relaxed and penalties for noncompliance with regulatory guidelines were disposed of, resulting in less scrutiny for physicians caring for out‐of‐state patients.^31^ The new legislation also allowed for the use of any remote communication product, provided it is not public‐facing, for medical communication, thereby attenuating the problem of patient access to technology as telemedicine can now be conducted via smartphone, which 85% of Americans own.^32^, ^33^

There remains a significant concern among physicians that when the temporary COVID‐19 expansions lapse, the lack of parity in reimbursement will end with them. Although private insurance providers and CMS have expressed intentions to continue equal reimbursement, concern for rising costs and fraud may prevent this action.^30^ There are currently several bills for the expansion of telehealth reimbursement under consideration in various state legislatures and in Congress.^34^, ^35^ Although the decisions on these legislative measures have not been made, telehealth continues to be a juggernaut in healthcare so parity in reimbursement must be achieved.

The COVID‐19 pandemic has also altered the educational framework in surgical oncology. Respondents reported decreased involvement of residents and fellows in virtual appointments, which may detract from their education and readiness to practice independently. However, instruction via virtual platforms can be as effective as in‐person instruction as it pertains to attaining surgical skills and knowledge.^36^ Congruently, most institutions have moved their tumor board discussions to a virtual platform, which SSO members perceived as better or equivalent to having tumor board in person. The lack of geographic limitation allows for international collaboration.^8^ The society of endocrine surgery found that using virtual platforms helped physicians develop new skills, amass new knowledge, and get to know their peers better.^37^ Continuing national and international collaboration in surgical oncology is certain to lead to better patient management, innovative solutions, and uniform management guidelines.

There is uncertainty surrounding the impact of changing practices on future outcomes for surgical oncology patients. More research must be done on long‐term effects of increased use of neoadjuvant therapy and delayed operations on the survival rates and prognosis of these patients. A practice change which is likely permanent is the increased use of telemedicine, as 65.3% (77/118) of respondents foresee themselves using telemedicine in the future. If questions of reimbursement, adequate physical examination, and patient access to technology are addressed, then telemedicine may become a vital tool in the management of select surgical oncology patients.

This study has several limitations. Firstly, all responses were voluntary so overly negative or positive experiences could have influenced SSO members to respond thereby biasing the results. The study had a small sample size and low response rate indicating that it might not be representative of surgical oncology practice across the board. Additionally, most respondents were academic physicians and located in urban areas, therefore not reflective of telemedicine practices in rural and nonacademic settings. Most respondents were in the US, limiting the applicability of this study internationally.

## 5 CONCLUSIONS

In conclusion, this survey of SSO members aimed to assess the impact of the COVID‐19 pandemic on clinical practice. Surgeons reported an increase in delayed operations and in the use of neoadjuvant therapy. Additionally, surgeons noted an increased use of telemedicine, which may be appropriate for long‐term follow‐up visits and postoperative visits, but not for first‐time patients. Challenges regarding reimbursement, patient access to technology, and performing an adequate physical examination remain. Future studies will examine the long‐term impact of changed practices on surgical oncology patients and reevaluate the place of telemedicine in surgical oncology.

## CONFLICTS OF INTEREST

The authors declare no conflicts of interest.

## SYNOPSIS

A survey of SSO members found the COVID‐19 pandemic resulted in more frequent neoadjuvant therapy, procedural delays, and telemedicine. Telemedicine was most efficacious for surveillance or postoperative visits, while in‐person visits were preferred for new patients.

## Data Availability

Data are available from the corresponding author upon reasonable request.
